# High-throughput design of high-performance lightweight high-entropy alloys

**DOI:** 10.1038/s41467-021-24523-9

**Published:** 2021-07-15

**Authors:** Rui Feng, Chuan Zhang, Michael C. Gao, Zongrui Pei, Fan Zhang, Yan Chen, Dong Ma, Ke An, Jonathan D. Poplawsky, Lizhi Ouyang, Yang Ren, Jeffrey A. Hawk, Michael Widom, Peter K. Liaw

**Affiliations:** 1 Department of Materials Science and Engineering, The University of Tennessee, Knoxville, TN USA; 2 Neutron Scattering Division, Oak Ridge National Laboratory, Oak Ridge, TN USA; 3 Computherm, LLC, Middleton, WI USA; 4 National Energy Technology Laboratory, Albany, OR USA; 5 Leidos Research Support Team, Albany, OR USA; 6 ORISE, 100 ORAU Way, Oak Ridge, TN USA; 7 Neutron Science Platform, Songshan Lake Materials Laboratory, Dongguan, Guangdong China; 8 Center for Nanophases Materials Sciences, Oak Ridge National Laboratory, Oak Ridge, TN USA; 9 Department of Physics and Mathematics, Tennessee State University, Nashville, TN USA; 10 Advanced Photon Source, Argonne National Laboratory, Argonne, IL USA; 11 Department of Physics, Carnegie Mellon University, Pittsburgh, PA USA

**Keywords:** Phase transitions and critical phenomena, Structural materials, Metals and alloys, Computational methods

## Abstract

Developing affordable and light high-temperature materials alternative to Ni-base superalloys has significantly increased the efforts in designing advanced ferritic superalloys. However, currently developed ferritic superalloys still exhibit low high-temperature strengths, which limits their usage. Here we use a CALPHAD-based high-throughput computational method to design light, strong, and low-cost high-entropy alloys for elevated-temperature applications. Through the high-throughput screening, precipitation-strengthened lightweight high-entropy alloys are discovered from thousands of initial compositions, which exhibit enhanced strengths compared to other counterparts at room and elevated temperatures. The experimental and theoretical understanding of both successful and failed cases in their strengthening mechanisms and order-disorder transitions further improves the accuracy of the thermodynamic database of the discovered alloy system. This study shows that integrating high-throughput screening, multiscale modeling, and experimental validation proves to be efficient and useful in accelerating the discovery of advanced precipitation-strengthened structural materials tuned by the high-entropy alloy concept.

## Abstract (web summary)

Advanced screening strategies for the design of high-entropy alloys are highly desirable. Here the authors use the project-oriented design strategy and CALPHAD-based high-throughput calculation tool to rapidly screen promising Al-Cr-Fe-Mn-Ti structural HEAs for high-temperature applications.

## Introduction

The fields of aerospace and fossil energy have benefited from decades of the development of Ni-base superalloys. However, to obtain further efficiency gains and environmental friendliness, developing new materials with inexpensive, light, and strong characteristics is required. Numerous efforts can be found in the development of novel ferritic superalloys containing a disordered body-centered-cubic (BCC) matrix with ordered B2 and/or L2_1_ precipitates due to their good creep resistance at elevated temperatures and relatively lower densities^1,2^. However, these emerging precipitation-strengthened ferritic alloys still face some drawbacks, such as the low high-temperature strength and not lightweight, which limit their applications. To overcome these issues, new alloy-design strategies are required. Recently, the concept of high-entropy alloys (HEAs) or multi-principal-element alloys (MPEAs) has revolutionized the traditional alloy-design strategy, using multi-principal components (≥5) instead of one or two key components^3–10^. Due to the existence of many kinds of elements with different atomic sizes, atoms in HEAs tend to deviate from their ideal lattice sites and give rise to severe local lattice distortion^5,6^, which could impede dislocation motion, leading to the pronounced strengthening effect^11,12^. To pursue higher strengths, the formation of coherent intermetallic precipitates while maintaining a medium- to high-entropy matrix has been attempted during the design of HEAs^10,13–15^. Moreover, the low lattice misfit that can decrease the nucleation barrier for precipitation, and thus stabilize precipitates with a high number density is likely found in HEAs^10,16^. These effects have demonstrated the concept of HEAs being a new avenue for developing new-type precipitation-strengthened lightweight and low-cost materials for high-temperature applications^17,18^. In the development of such materials, critical goal-oriented design strategies are the prerequisite, including the inexpensive raw materials, low density, high melting temperature (T_m_), good oxidation resistance, great creep resistance, and high strength with acceptable ductility. Thus, a rational selection of chemical compositions is of very significance to take into account these essential factors. Our previous work discovered potential L2_1_ precipitation-strengthened lightweight HEAs (LWHEAs) in an Al–Cr–Fe–Mn–Ti system, but the formation of the brittle C14 Laves phase deteriorates their properties because the dissimilarity of crystal structures and different thermal expansion coefficients between the C14 Laves and BCC-base phases increase the tendency to crack^18^. Hence, exploring suitable chemical compositions without the formation of detrimental intermetallic phases is crucial for developing the desired materials. However, the vastness of the compositional space poses a huge challenge for efficiently screening out suitable compositions. The trial-and-error experimental method is obviously not suitable, since it is extremely costly and time-consuming to experimentally screen the proper alloys in the vast composition space. This trend is even true for a quinary system, not to mention a higher-order (*n* ≥ 5) multicomponent system, like the Al–Cr–Fe–Mn–Ti system. Fortunately, the pace of discovering promising HEAs can, in principle, be accelerated by the development of efficient computational screening methods and tools^19,20^. In the present work, the CALculation of PHAse Diagrams (CALPHAD)-based high-throughput computational tool is employed to efficiently explore the Al–Cr–Fe–Mn–Ti system for discovering new-type precipitation-strengthened HEAs. The subsequent experimental and theoretical studies on the discovered lightweight HEAs provide insights into developing high-performance HEAs via the high-throughput alloy-design method.

## Results

### CALPHAD-based high-throughput calculation (HTC) screening results

The goal of the current work is to design high-performance LWHEAs for elevated-temperature applications. Since materials in use at high temperatures approach equilibrium states, knowledge of stable phase equilibrium at fabrication and working temperatures are very important. Miracle and Senkov et al.^21,22^ have explored the development of multi-principal element alloys for structural applications and have summarized a few criteria as guidelines for the design of high-temperature structural materials:

1. No first-order phase transformation in the temperature range of the application is allowed to ensure structural stability. Therefore, all phase transformations, if present, must be above the operating temperature, *T*_use_ (about 0.8*T*_m_, where the temperatures are in Celsius degree).
2. Good ductility and fracture toughness are required. Therefore, the microstructure must include a ductile solid-solution primary phase as the matrix and a precipitation-strengthening phase that can dissolve at high temperatures and then re-precipitate above *T*_use_ but below *T*_m_.

Our previous research on the Al–Cr–Fe–Mn–Ti HEAs^18^ reported the formation of high-density coherent L2_1_ precipitates in a disordered BCC solid-solution matrix. Therefore, the L2_1_ phase is chosen as the target strengthening-precipitate phase. The optimal molar fraction of the L2_1_ phase is not known. Hence, we set it to be 0.05–0.5 at the application temperatures as a reasonable approximation. The specific criteria used for the present HTCs are

(I) *T*_m_ > 1250 °C and f(BCC) = 1 at *T*_m_;
(II) *f*(BCC) + *f*(L2_1_) = 1 and 0.05 <  *f*(L2_1_) < 0.5 at 0.8*T*_m_;
(III) *f*(BCC) + *f*(L2_1_) = 1 and 0.05 <  *f*(L2_1_) < 0.5 at 0.5*T*_m_.

**Fig. 1:**
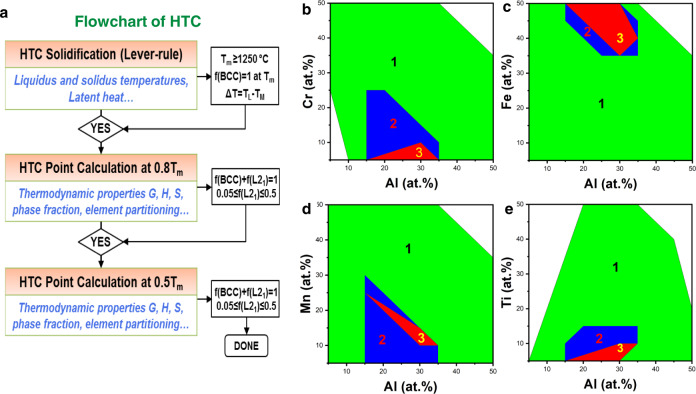
High-throughput screening optimal alloy compositions in the Al–Cr–Fe–Mn–Ti system. **a** Flowchart of the current HTC. **b** Al–Cr projection. **c** Al–Fe projection. **d** Al–Mn projection. **e** Al–Ti projection.

Here *f*(BCC) and *f*(L2_1_) refer to the mole fractions of the BCC and L2_1_ phases, respectively. The first criterion ensures that the primary phase is the disordered BCC solid solution. The second and third criteria ensure that the L2_1_ phase is the precipitation-strengthening phase and no phase transformation in the temperature range for target applications (0.5*T*_m_ < *T* < 0.8*T*_m_). The HTC flowchart and results are summarized in Fig. 1. In Fig. 1b–e, Area 1 only meets criterion I. Area 2 meets criteria I and II, and Area 3 meets all three criteria. Using a step size of 5 atomic percent (at.%) for each component in the composition range from 0 to 50 at.%, the solidification HTCs for a total of 3246 alloys are calculated, using the lever-rule model. From this first-round HTC, the melting temperature (*T*_m_) of each alloy as well as its phase stability at *T*_m_ can be obtained. Two criteria are used to screen our target alloys: *T*_m_ > 1250 °C and a single BCC phase at *T*_m_. One third of these alloys (1168) are found to meet the criterion (Area 1). In the second round, the 0D point HTCs are carried out for the 1168 alloys at 0.8*T*_m_, and the criterion of *f*(BCC) + *f*(L2_1_) = 1 and 0.05 < *f*(L2_1_) < 0.5 is as the filter. A total of 44 alloys are identified and shown as Area 2 in Fig. 1b–e. In the third round, the 0D point HTCs are performed for these 44 alloys at 0.5*T*_m_ and the same criterion of (BCC) + *f*(L2_1_) = 1 and 0.05 < *f*(L2_1_) < 0.5 is used as the filter. Only eight alloys (density, *ρ* ≤ 6.5 g/cm^3^) survive out of the over 3000 compositions eventually. The compositions that meet all three criteria (I, II, and III) are shown as Area 3 in Fig. 1b–e. Detailed compositions and the calculated equilibrium phase of all identified alloys are listed in Supplementary Table 1 and Supplementary Fig. 1, respectively.

### Microstructural and mechanical validations on the discovered LWHEAs

Systematic experimental investigations on the identified eight LWHEAs were then carried out not only to validate the CALPHAD-based HTC predictions, but also to obtain essential data (microstructures and mechanical properties) and valuable insights for improving the accuracy of high-throughput predictions further.

**Fig. 2:**
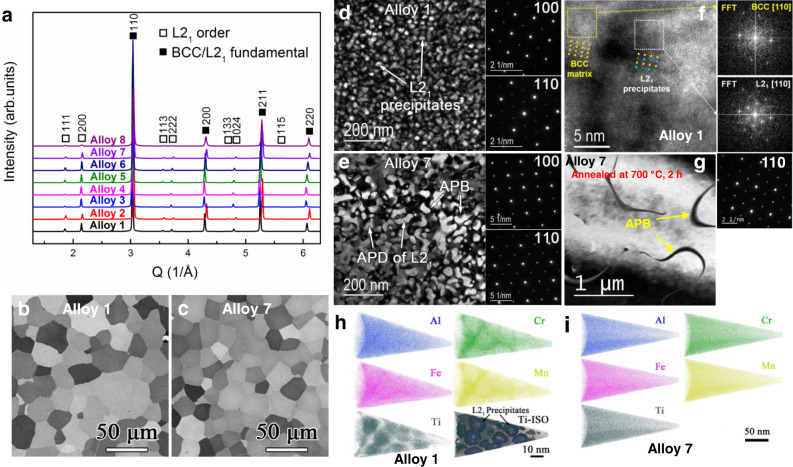
Microstructural information of the discovered LWHEAs. **a** Synchrotron XRD patterns of Alloys 1–8 in their as-cast states. **b**, **c** Backscattered electron (BSE) images of the representative Alloys 1 (Al_20_Cr_5_Fe_50_Mn_20_Ti_5_) and 7 (Al_30_Cr_5_Fe_50_Mn_10_Ti_5_), representatively. **d**, **e** The TEM DF images taken by the unique (111) reflection of the L2_1_ phase and the corresponding selected-area diffraction (SAED) patterns along the [100] and [110] zone axes of Alloys 1 and 7 in their as-cast states, respectively. The arrows indicate the features of precipitates, antiphase domain (APD), and antiphase boundary (APB). **f** High-resolution TEM (HRTEM) image of Alloy 1 taken along the [110] zone axis, showing the coherent interfaces between the BCC matrix and the L2_1_ nanoscale precipitates. The next two images are the fast Fourier Transform (FFT) patterns of the BCC matrix and L2_1_ precipitates of Alloy 1. **g** The TEM DF images taken by the unique (111) reflection of the L2_1_ phase of the Alloy 7 after annealing at 700 °C for 2 h, showing the greatly grown APDs. **h**, **i** APT atomic maps of Alloys 1 and 7, respectively.

**Fig. 3:**
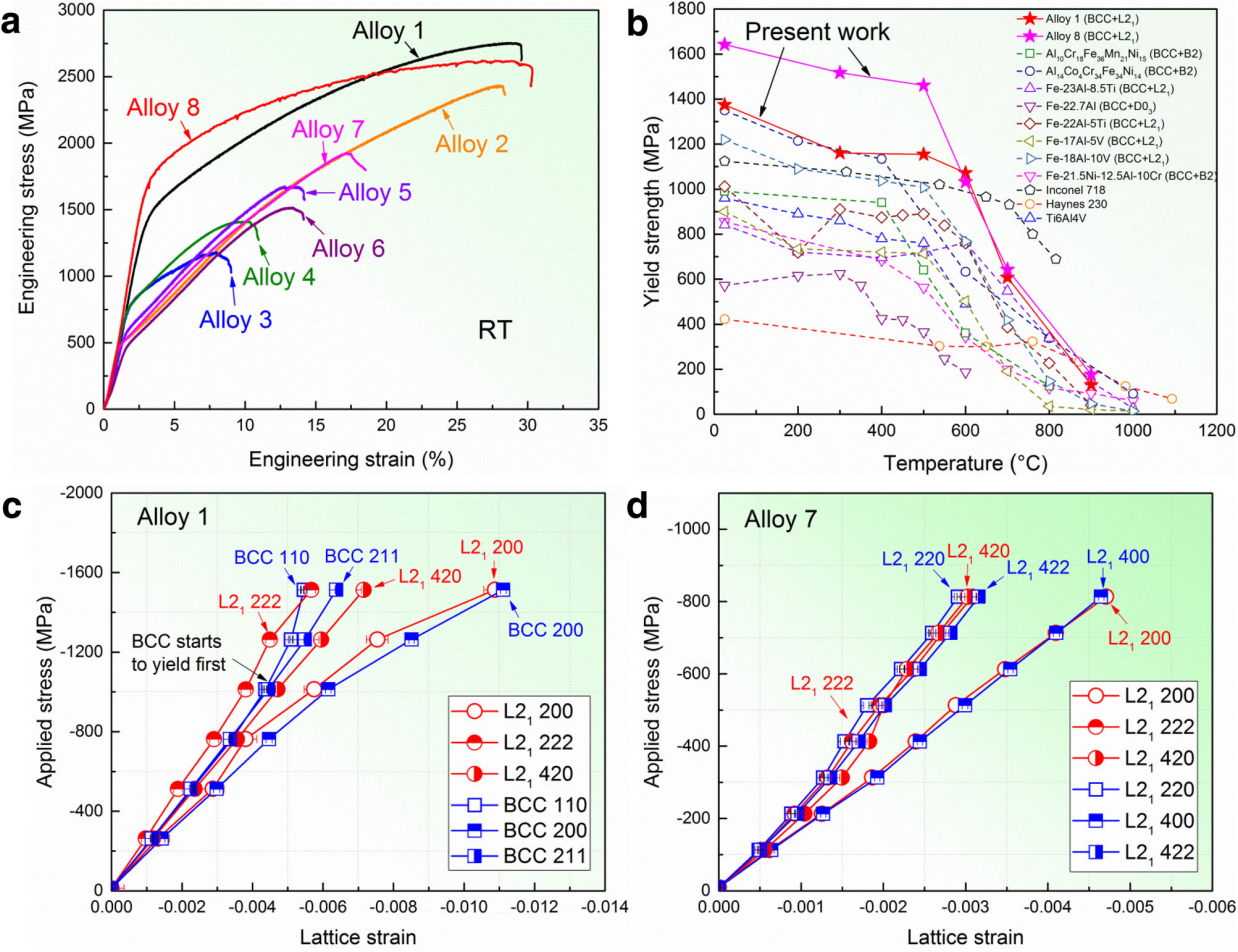
Mechanical responses of the discovered LWHEAs. **a** Compressive stress-strain plots of Alloys 1–8 at RT. **b** Comparison of yield strengths as a function of temperature between Alloys 1, 8, and other counterpart materials (all these BCC-base alloys are in their as-cast states)^23–27^. **c**, **d** Lattice strain as a function of applied stress along the loading direction in Alloys 1 and 7 at RT, respectively, measured by in situ neutron diffraction. The error bars are obtained from the uncertainties of the single-peak fitting on *hkl* diffraction peaks.

The synchrotron x-ray diffraction (XRD) patterns (Fig. 2a) demonstrate that all the eight alloys in the as-cast states are composed of L2_1_ and possible BCC phases without other intermetallic phases. All the eight alloys have equiaxed grains (Fig. 2b, c and Supplementary Fig. 2), indicative of a single-phase structure. The morphologies of the L2_1_ phase in these LWHEAs were characterized by the transmission-electron microscopy (TEM) dark-field (DF) images (Fig. 2d, e and Supplementary Fig. 3). Interestingly, two different L2_1_ morphologies are present among the eight alloys. The high-density nanoscaled L2_1_ precipitates (10–30 nm) that are uniformly distributed within the BCC matrix are found in Alloy 1 (Al_20_Cr_5_Fe_50_Mn_20_Ti_5_) and Alloy 8 (Al_15_Cr_5_Fe_50_Mn_25_Ti_5_) (Fig. 2d and Supplementary Fig. 3), while the typical ordered L2_1_ antiphase domains (APDs) with a thin film of a disordered BCC phase on the antiphase domain boundaries (APBs) are observed in Alloys 2–7 (Fig. 2e and Supplementary Fig. 3). In addition to the as-cast states, we also performed the TEM characterizations on the representative Alloys 1, 2, and 7 after homogenization at 1200 °C (Supplementary Fig. 4). For Alloy 1, its annealed microstructure is very similar to that in the as-cast state (Fig. 3d), but with a growth of the L2_1_ precipitates to ~39 nm (Supplementary Fig. 4a). For Alloys 2 and 7, the sizes of APD are greater than those in the as-cast condition (Supplementary Fig. 4b, c), indicating that the L2_1_ phase is stable at 1200 °C. To further demonstrate the L2_1_ phase’s stability at intermediate temperatures, we also annealed the as-cast Alloy 7 at 700 °C for 2 hours. As shown in Fig. 2g, the sizes of L2_1_ APD grew greatly, suggesting that the alloys with an L2_1_ APD morphology are more like a single L2_1_ phase below its melting point.

The chemical compositions and morphologies of the constituent phases in the representative Alloys 1 and 7 were determined by atom-probe tomography (APT) (Fig. 2h, i). In Alloy 1, the L2_1_ phase is rich in Al and Ti, while the BCC phase contains mostly Cr, Mn, and Fe (Fig. 2h and Table 1). A similar compositional distribution in the L2_1_ and BCC phases is also observed in Alloy 8 (Table 1). Moreover, the L2_1_ number density (2.76 × 10^22^/m^3^) and volume fraction (43.6 volume percent, vol.%) (~44 vol.% by the Rietveld refinement, Supplementary Fig. 5) in Alloy 1 were also determined by the APT analysis. Note that the number density is highly underestimated because precipitates are touching or overlapping so that they cannot be delineated from each other, using isosurfaces. In contrast to Alloy 1, the chemical distribution of constituent elements in Alloy 7 is uniform (Fig. 2i). Together with the TEM observations (Fig. 2e), it suggests that Alloy 7 consists of ordered L2_1_ APDs separated by APBs, and no chemical segregation is found between the APDs and the APBs.

**Table 1:** Chemical compositions of the BCC and L2_1_ phases in Alloys 1 and 8, measured by APT.

*Alloy 1 (Al*_*20*_*Cr*_*5*_*Fe*_*50*_*Mn*_*20*_*Ti*_*5*_*)*					
Phase	Al (at.%)	Cr (at.%)	Fe (at.%)	Mn (at.%)	Ti (at.%)
BCC	16.63 ± 0.09	6.70 ± 0.05	53.10 ± 0.10	22.79 ± 0.09	0.79 ± 0.02
L2_1_	23.67 ± 0.14	3.21 ± 0.06	49.82 ± 0.14	11.84 ± 0.11	11.46 ± 0.10
*Alloy 8 (Al*_*15*_*Cr*_*5*_*Fe*_*50*_*Mn*_*25*_*Ti*_*5*_*)*					
Phase	Al (at.%)	Cr (at.%)	Fe (at.%)	Mn (at.%)	Ti (at.%)
BCC	13.31 ± 0.04	6.04 ± 0.01	53.09 ± 0.05	26.35 ± 0.04	1.21 ± 0.01
L2_1_	22.14 ± 0.09	2.62 ± 0.02	49.49 ± 0.10	10.40 ± 0.06	15.32 ± 0.07

To evaluate the mechanical properties of the identified eight alloys in a high-throughput manner, we performed compression tests on the eight alloys at room temperature (RT) (Fig. 3a). One can see that the yield strengths of these alloys vary in a wide range from 500 to 1642 MPa (Supplementary Table 2). The high-temperature performances of Alloys 1 and 8 were also evaluated and compared to other representative alloys (Fig. 3b, Supplementary Fig. 6, and Supplementary Table 3). Alloys 1 and 8 exhibit high yield strengths below 700 °C (1072 ± 25 MPa at 600 °C for Alloy 1; 1032 ± 10 MPa at 600 °C for Alloy 8). As the temperature increases to 700 °C, the yield strengths of Alloys 1 and 8 are still kept at 607 ± 22 MPa and 643 ± 16 MPa, respectively, though an obvious softening occurs like many other precipitation-strengthened ferritic BCC alloys^23–28^. However, the newly designed L2_1_ precipitation-strengthened LWHEAs outperform traditional precipitation-strengthened BCC alloys, such as BCC + L2_1_, BCC + D0_3_, and BCC + B2 alloys, in terms of yield strengths and specific yield strengths (Fig. 3b and Supplementary Fig. 7). Moreover, the softening resistance in these alloys above 700 °C could be further improved by the alloy-design wisdom in Ni-base superalloys, such as the addition of minor refractory elements, e.g., W, Ta, and Mo^29^.

Based on the above experimental analyses, we can categorize the eight alloys into two groups. G1: Alloys 1 and 8 with the BCC matrix + nanoscale L2_1_ precipitates (Fig. 2 and Supplementary Fig. 3), in line with our thermodynamic prediction. G2: Alloys 2–7 with the L2_1_ as the major phase (Fig. 2 and Supplementary Fig. 3), in which the L2_1_ phase fractions are too high and deviate from our thermodynamic prediction. For easy understanding, our discussion in the following part will base on these two groups.

### Strengthening behavior

The different mechanical responses between G1 (Alloys 1 and 8) and G2 (Alloys 2–7) are attributed to the precipitation strengthening and the intrinsic-deformation behavior of L2_1_, as dispersed particles or a single phase, respectively, considering L2_1_’s different morphologies and volume fractions (e.g., Alloy 1: precipitates, 44 vol.%; Alloy 7: APDs, ~85 vol.%). The precipitation-strengthening contribution in Alloy 1 is quantitatively calculated (Supplementary Note 1), which is mainly attributed to the ordering strengthening (~904 MPa), close to the yield strength differences between Alloys 1 and 2 (~874 MPa), and between Alloys 1 and 7 (~860 MPa). The precipitation-strengthening effect in Alloy 1, but not in Alloy 7, is also demonstrated by the in situ neutron-diffraction results. Figure 3c, d exhibit the lattice strain versus applied compressive stress curves corresponding to the loading direction in both Alloys 1 and 7. One can notice that Alloy 1 displays a clear load-transfer behavior upon the BCC phase yielding (around 1000 MPa), i.e., the hard L2_1_ phase bears a larger load with greater lattice strains (L2_1_ {222} and {420}), while the soft BCC phase undertakes a lower load with lower lattice strains (BCC {110} and {211}), which is a reflection of the precipitation strengthening. In contrast, no load transfer was observed in Alloy 7 (Fig. 3d). The synchronized response of various *hkl*-specific lattice strains versus applied stress indicates that Alloy 7 behaves like a single L2_1_ phase. Therefore, the high yield strengths of Alloys 1 and 8 are attributed to the precipitation strengthening, besides the effect of atomic-level complexity in HEAs.

### Order-disorder transition behavior

**Fig. 4:**
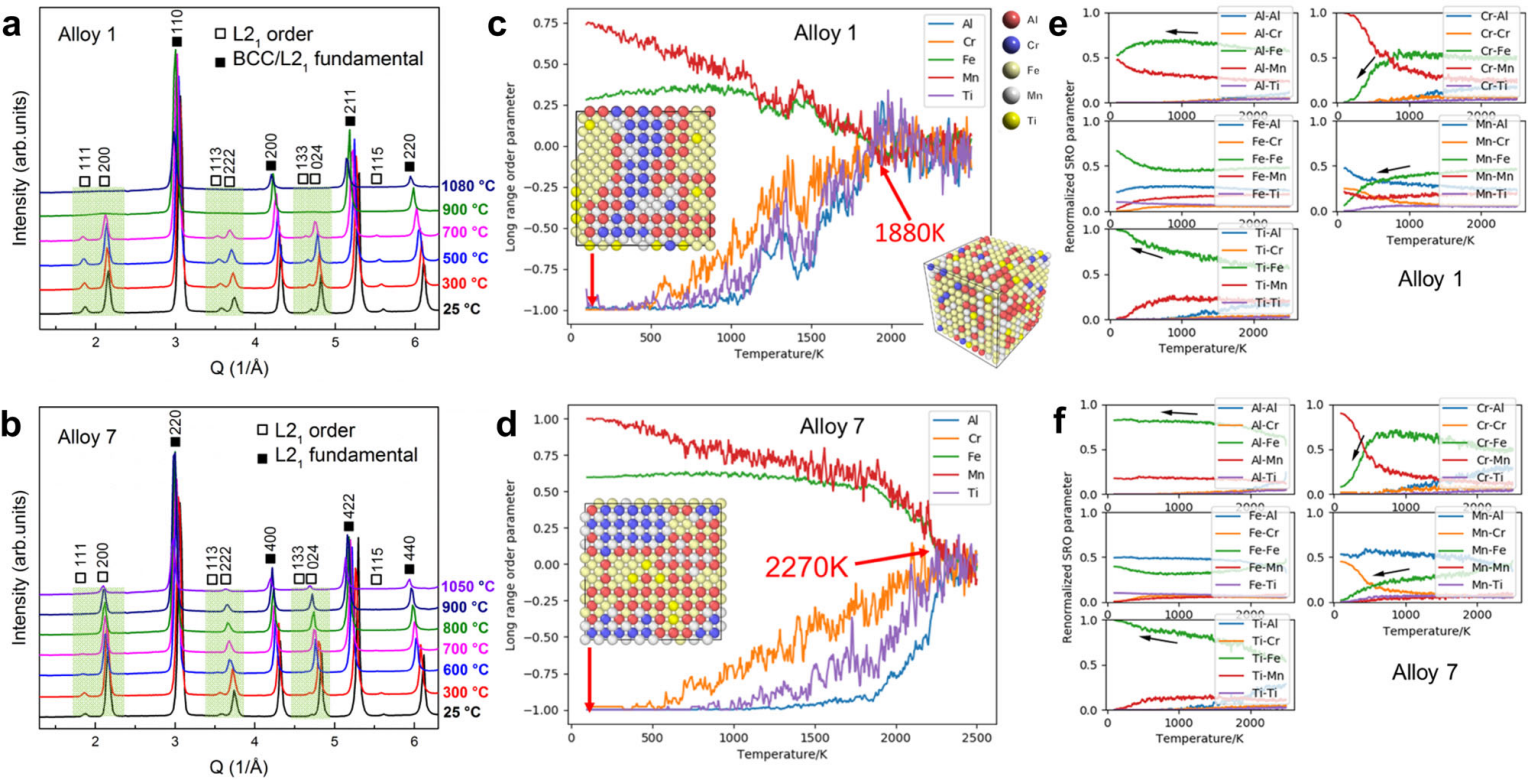
Order-disorder transitions of Alloys 1 and 7. **a**, **b** In situ neutron-scattering patterns of Alloys 1 (Al_20_Cr_5_Fe_50_Mn_20_Ti_5_) and 7 (Al_30_Cr_5_Fe_50_Mn_10_Ti_5_) at elevated temperatures, respectively. The label of order means that the reflections are exclusive to the L2_1_ structure, while the label of fundamental means that the reflections are common to both the ordered L2_1_ and disordered BCC structures. **c**, **d** The MC-calculated LRO parameters as a function of temperature in Alloys 1 and 7, respectively. **e**, **f** The MC-calculated SRO parameters as a function of temperature in Alloys 1 and 7, respectively. The black arrows indicate the change of the most abundant, Fe, as the neighbor of all elements.

The morphology of the L2_1_ phase strongly affects the mechanical performance of the high-throughput-designed LWHEAs, which is related to the order-disorder transition behavior. Thus, we performed the in situ neutron scattering on the representative Alloy 1 (Al_20_Cr_5_Fe_50_Mn_20_Ti_5_) in G1 and Alloy 7 (Al_30_Cr_5_Fe_50_Mn_10_Ti_5_) in G2 to investigate this behavior. As shown in Fig. 4a for Alloy 1, the unique L2_1_ peaks (highlighted by the green color) are observed only at 900 °C and below, indicating that L2_1_ is stable up to 900 °C, below which both the BCC and L2_1_ phases coexist. The relative intensity of superstructure peaks for the L2_1_ phase decreases upon heating, which is due to the reduced fraction of the L2_1_ phase. For Alloy 7, the order peaks of the L2_1_ phase persist to 1050 °C, suggesting that L2_1_ exists even up to 1050 °C (Fig. 4b and Supplementary Fig. 8).

We further conducted the metropolis Monte Carlo (MC) calculations to understand the order-disorder transitions in Alloys 1 and 7 during solidification, using much larger supercells of 2000 atoms at 10^7^ MC steps within the nearest-neighbor interaction approximation. With the concentrations as the only variables for a given system, this method gives a more clear and definitive picture of the influence of constitutions on the order-disorder transition. The output of the MC calculations allows us to calculate the short-range ordering (SRO) and long-range ordering (LRO) parameters for both Alloys 1 and 7 between 0 and 2500 K. Define ${\xi }_{\mu }^{i}=1$when a site, *i*, is occupied by an atom of a type, μ, and ${\xi }_{\mu }^{i}=0$ otherwise. A configuration is uniquely specified by the set, $\{{\xi }_{\mu }^{i}\}$. Note the identities, ${\varSigma }_{\mu }{\xi }_{\mu }^{i}=1$ and $< {\xi }_{\mu }^{i} > ={c}_{\mu }$. The frequency of the nearest-neighbor pairs of species, μ and ν, is${y}_{\mu \nu }^{ij}= < {\xi }_{\mu }^{i}{\xi }_{\nu }^{j} >$. We define

1 $${w}_{\mu \nu }^{ij}\equiv {y}_{\mu \nu }^{ij}/{c}_{\mu }= < {\xi }_{\mu \nu }^{ij}{\xi }_{\nu \nu }^{ij} > /{c}_{\mu }$$

As the conditional probability that a site, *j*, has species, *ν*, given that *i* has species, *μ*. Our ${w}_{\mu \nu }^{ij}$is related to the usual Warren-Cowley order parameter^30,31^ as ${\alpha }_{uv}^{ij}=1-{w}_{\mu \nu }^{ij}/{c}_{\mu }$.

The L2_1_ structure has three different sublattice sites (Wyckoff sites): 8c, 4a, and 4b, in which 4a and 4b can be considered as one site and 8c as the other site. Therefore, for simplification, we can treat an L2_1_ structure as a B2 structure to calculate the LRO parameter. The LRO can be expressed as^32^

2 $${\eta }_{i}=\frac{{y}_{i}^{\alpha }-{y}_{i}^{\beta }}{{y}_{i}^{\alpha }+{y}_{i}^{\beta }}$$

where ${y}_{i}^{\alpha }$ and ${y}_{i}^{\beta }$are the site occupancy of the element, *i*, on sublattices, *α* and *β*, respectively. The value of ${\eta }_{i}$ falls within the range of −1 to 1, as shown in Fig. 4c, d. When ${\eta }_{i}=0$, the element, *i*, is randomly distributed on both *α* and *β* sublattices. When ${\eta }_{i}$ equals 1 or −1, the element, *i*, can be exclusively found on the *α* or *β* sublattice.

The order-disorder transition can be directly reflected by the LRO parameters (Fig. 4c, d). In both Alloys 1 and 7, as the temperature decreases, Al, Cr, and Ti tend to segregate to one sublattice, while Fe and Mn primarily occupy the other. As indicated by the red arrows, the ordering transition temperature, *T*_c_, of Alloy 1 [1607 °C (1880 K)] is much lower than that of Alloy 7 [1997 °C (2270 K), far beyond its melting temperature], suggesting that the ordered L2_1_ phase is less easily retained in the solid state in Alloy 1 than in Alloy 7^33^. This prediction is consistent with the experimental observations (Fig. 4a, b), i.e., Alloy 1 is composed of L2_1_ and BCC phases from RT to 900 °C, while only the ordered L2_1_ phase still exists in Alloy 7 from RT to 1050 °C. The inset subfigures give the snapshots at −173 °C (100 K), which directly show the L2_1_-phase formation. Besides the LRO, the SRO parameters reveal more details of the order-disorder transition (Fig. 4e, f). Since Fe is the most abundant element in this system, we consider its changes as the fingerprint of the ordering transition. In both alloys, the temperature-dependent SRO parameters of Fe atoms as the nearest neighbors of Al and Ti increase upon cooling. In contrast, the Cr and Mn have the opposite trend (Fig. 4e, f). This trend is particularly obvious for Ti that has only Fe as its neighbors around and below 727 °C (1000 K). Moreover, the SRO parameters also demonstrate that the formation of the L2_1_ phase is favored by Fe–Al and Fe–Ti pairs and that of the BCC phase by Cr–Fe pairs, in agreement with the chemical compositions of L2_1_ and BCC phases (Fig. 2h and Table 1). The reliability of the MC 2000-atom superstructures for both Alloys 1 and 7 are verified by the fitted neutron-scattering pair-distribution function (PDF) results (Supplementary Fig. 9) and by comparison with the result of first-principles simulations (Supplementary Table 4).

To better show the ordering transition due to the formation of the Fe_2_AlTi-type L2_1_ phase, we performed another MC simulation with 250 atoms at 10^6^ MC steps especially for this system between −273 and 2727 °C (0–3000 K) (Supplementary Figs. 10, 11). The ordered L2_1_ structure starts to become less ordered at ~ 1227 °C (1500 K). Both the LRO and SRO disappear at about 2327 °C (2600 K). Through comparing the LRO and SRO between the Fe_2_AlTi-type L2_1_ and Alloys 1 and 7, we can find that the ordering temperature in Alloy 7 with a higher Al content is closer to that of the Fe_2_AlTi-type L2_1_ structure, implying that the Fe_2_AlTi-type L2_1_ can survive at higher temperatures in Alloy 7 than Alloy 1, in line with the in situ neutron-scattering results (Fig. 4a, b).

**Fig. 5:**
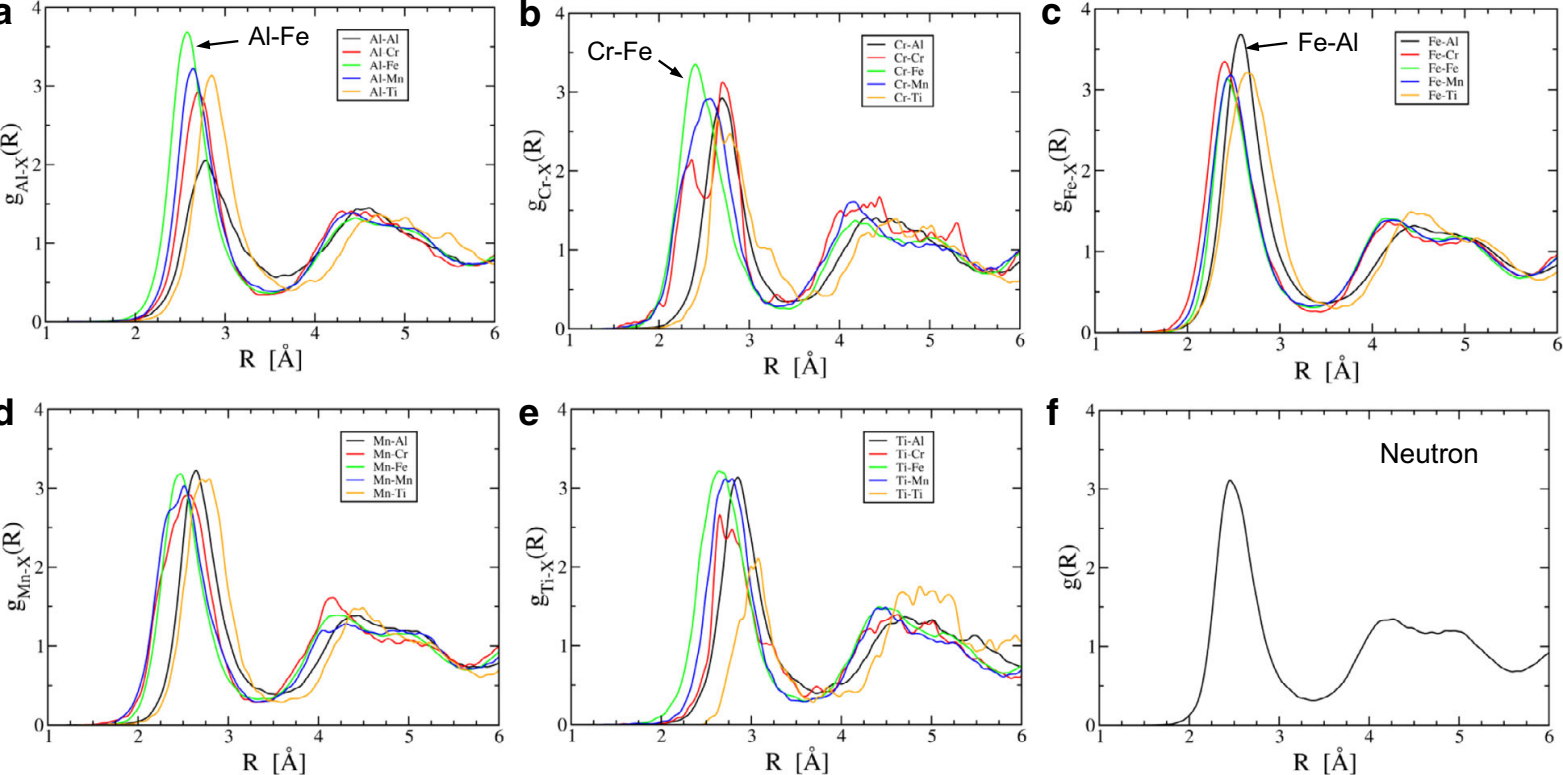
First-principles calculations on Alloy 1. **a**–**e** AIMD-simulated partial PDFs of Alloy 1 (Al_20_Cr_5_Fe_50_Mn_20_Ti_5_) at *T* = 1600 °C, and **f** Neutron-weighted PDF.

Ab initio molecular dynamics (AIMD) simulations were also performed on Alloy 1 in its molten state to support the MC-calculation results. Figure 5a–e shows the selected partial PDF of Alloy 1 at *T* = 1600 °C to the first nearest neighbor. The intensities of pair correlations of the Al–Al and Ti–Ti bonds are significantly lower than the other pair correlations, indicating the strong tendency for Al and Ti atoms to bond with other elements. On the other hand, the Al–Fe pair correlation has the highest intensity, and the Cr–Fe pair correlation has the second-highest intensity, compared to other pair correlations. The persistent presence of stronger Al–Fe and Cr–Fe pair correlations in the Al–Cr–Fe–Mn–Ti alloys suggests that Al–Fe and Cr–Fe pair interactions may promote the formation of L2_1_ and BCC phases, respectively, as evidenced by the chemical compositions of both phases (Table 1).

### Reason for the formation of different L2_1_ morphologies

**Fig. 6:**
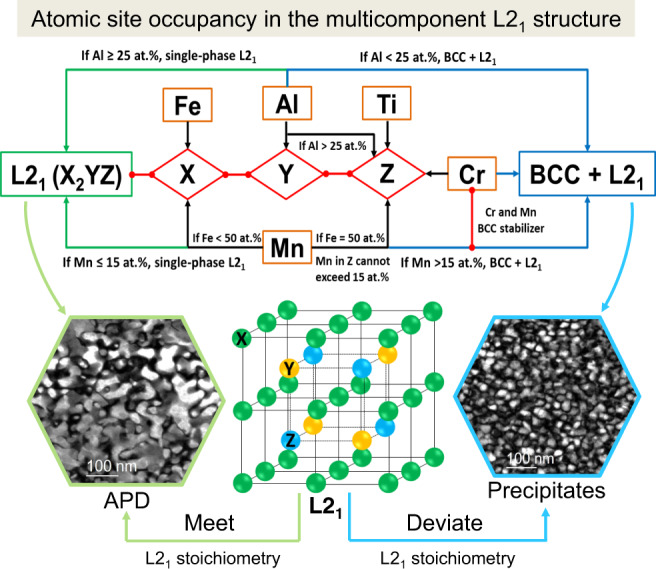
Schematic of the atomic-site occupancy and morphology evolution of the L2_1_ structure. A schematic showing the atomic-site occupancy in the multicomponent L2_1_ structure and the morphology evolution of the L2_1_ phase that depends on the chemical compositions of discovered LWHEAs.

The different L2_1_ morphologies exhibited between G1 (nanoscale precipitates) and G2 (APDs) are strongly correlated to their different chemical compositions (Supplementary Table 1), which directly affect their order-disorder transitions, namely, the atomic-site occupancy of the constituent elements. The atomic-site occupancy of the formed multicomponent L2_1_ structure is revealed as follows. The L2_1_ (X_2_YZ) structure (Prototype, Fe_2_AlTi; Pearson type, cF16) has three different sublattice sites: X @ 8c(1/4, 1/4, 1/4), Y @ 4a(0, 0, 0), and Z @ 4b(1/2, 1/2, 1/2). According to the calculated LRO parameters (Fig. 4c, d), Fe and Mn prefer to occupy one sublattice, while Al, Ti, and Cr tend to segregate into another sublattice. In the L2_1_ structure, the sublattices of Y and Z can be considered equally in terms of site, but differ in terms of composition. Taking account of the strong atomic pairs of Al–Fe, Fe–Ti, Fe–Cr, and Ti–Al (Fig. 5), we can assert that Y(4a) and Z(4b) are filled with Al and (Ti, Cr), respectively. However, if the content of Al is more than 25 at.%, the extra Al atoms can also occupy the site of Z(4b). In the L2_1_ structure of the present Al–Cr–Fe–Mn–Ti system, Fe atoms are always in the site of X(8c) because the content of Fe is not beyond 50 at.%. As for Mn, its site occupancy depends on Fe contents, that is, if the Fe content is less than 50 at.%, Mn can occupy the unfilled sites of X(8c) by Fe because Mn–Al and Mn–Ti have comparable interaction energies with Fe–Al and Fe–Ti, respectively (Table 2). Once the Fe content equals 50 at.%, the sites of X(8c) are completely by Fe atoms, and thus, Mn can occupy the unfilled sites at Z(4b) by Ti and Cr. Similar atomic occupancy of Mn at Z(4b) can be found in a Fe_2_AlMn L2_1_ structure^34^. Note that the solubility of Mn at sites of Z(4b) should not exceed 15 at.% because the Mn content in L2_1_ is always less than 15 at.% (Table 1 and Supplementary Table 1). When the content of Mn is more than 15 at.%, the extra Mn goes to the BCC matrix. Cr tends to segregate with Fe and Mn together in the disordered BCC matrix, as evidenced by the observed chemical composition in the BCC matrix of Alloys 1 and 8 (Table 1). From the known atomic-site occupancy, the different L2_1_ morphologies between G1 and G2 can be understood from the L2_1_ stoichiometric composition, i.e., ${(50)}_{{\rm{Fe}}({\rm{Mn}})}^{X}{(25)}_{{\rm{Al}}}^{Y}{(25)}_{{\rm{Ti}},{\rm{Cr}}\,({\rm{Al}},{\rm{Mn}})}^{Z}$. For G2 (Alloys 2–7), their chemical compositions can exactly match the perfect L2_1_ stoichiometry, resulting in the formation of the single-phase L2_1_ phase in the form of APD. In contrast, for G1 (Alloys 1 and 8), their chemical compositions deviate far away from the perfect L2_1_ stoichiometry, leading to the formation of nanoscaled L2_1_ precipitates within the BCC matrix. The atomic-site occupancy in the multicomponent L2_1_ structure and the composition-dependent phase-evolution behavior are schematically presented in Fig. 6, which can be further integrated with our HTC frame to accelerate the discovery of advanced precipitation-strengthened LWHEAs.

**Table 2:** The nearest-neighbor interaction parameters, ${J}_{ij}$, in eV.

${J}_{{ij}}$	Al	Cr	Fe	Mn	Ti
Al	0	−0.0224	−0.0942	−0.0970	−0.1280
Cr	−0.0224	0	0.0210	−0.0163	−0.0043
Fe	−0.0942	0.0210	0	0.0094	−0.1181
Mn	−0.0970	−0.0163	0.0094	0	−0.0930
Ti	−0.1280	−0.0043	−0.1181	−0.0930	0

## Discussion

The concept of HEAs provides us an unprecedented degree of freedom in the design of advanced alloys with promising properties. For the rapid exploration of the vast compositional space and investigation of the composition and temperature effects on the microstructure, advanced HEA-design strategies and efficient tools are necessary. At present, a few advanced HEA-design strategies were reported by combining the physicochemical criteria, CALPHAD, data mining, and multi-objective optimization algorithm^21,22,35–37^, demonstrating that the CALPHAD method has played an important role in the design of advanced HEAs. There are two essential requirements to carry out calculations, using the CALPHAD method: computational software and database.

The computational software could significantly affect the efficiency of HTC since massive calculations are carried out. In the example of the Al–Cr–Fe–Mn–Ti quinary system, it takes only a few hours on a desktop computer to finish the HTCs (~$3\times {10}^{3}$) within the composition range of 5–50 at.% for each element and finally screen out eight potential alloys. However, if a step size of 1 at.% for this quinary system is used, the computational time will easily increase to a few thousands of hours, posing a challenge for a single personal computer. The computational time will be scaled up substantially with higher-order systems (the number of components > 5). Fortunately, the current HTC module can be easily converted to parallel calculations for efficiently utilizing the available computational resource, which is the target that we are working on. In addition, the CALPHAD-developed datasets are multi-dimensional and very large. Thus, the organization of the large volume of data will be especially critical for efficiently retrieving the requested subsets of the large datasets. At present, each calculation is stored separately in a workspace and organized by its calculation identity (ID). The calculation ID is usually the alloy composition, and the detailed information of each calculation is represented by the predefined features, such as the composition, temperature, phase fraction, etc. Note that we can generally include all kinds of thermochemical and thermophysical properties in an individual CALPHAD calculation. The users can easily access these results via their calculation IDs and efficiently retrieve the requested datasets through the customized features. Here, we would like to emphasize that any algorithms that are able to be ascribed to these features can be employed as the screening criteria for the CALPHAD-calculated datasets. Moreover, the current HTC tool allows users to customize the outputs of different types of CALPHAD calculations for the possible combination with other datasets for machine learning and the usage of other data mining tools.

On the other hand, the accuracy of the CALPHAD method depends on the reliability of the thermodynamic database. The microstructure discrepancy between the thermodynamic prediction and experimental observation indicates that our current thermodynamic database needs further improvement on the stability of the L2_1_ phase. In addition to the current experimental results, long-period annealing on these identified alloys and quantitative characterization will be necessary in order to improve our current thermodynamic database.

Our results on the discovered LWHEAs demonstrate the sensitivity of the nanoscaled L2_1_ precipitates-strengthened BCC microstructure to alloys’ chemical compositions. Therefore, the scrutiny of the alloys’ chemical compositions that are related to the critical ordering temperature and morphology of L2_1_ precipitates is required during alloy design. However, the current high-throughput predictions are valid in the composition range of Al < 25 at.% and Mn > 15 at.% with the unchanged contents of other elements. Especially, the HTC can become more efficient when it is coupled with the deciphered atomic occupancy of the multicomponent L2_1_ structure and its related phase-evolution rule. Therefore, the high-throughput design and the fundamental understanding of the discovered LWHEAs will accelerate the pace of discovering promising HEAs, especially for multicomponent systems.

## Methods

### The CALPHAD-based HTCs

The CALPHAD approach^38–40^ is currently the only method that can be used to obtain multicomponent phase diagrams with enough accuracy for practical applications without the need of the heavy experimental work^41^. This argument has made the CALPHAD method a key building block in the two largest driving forces in materials engineering today—ICME (Integrated computational materials engineering)^42^ and MGI (Materials Genome Initiative)^43^. The application of the CALPHAD method requires both the thermodynamic database to provide the Gibbs energies (as a function of pressure, temperature, and composition) for the individual phases and the computational software to calculate the equilibrium state by an energy-minimization procedure. Over the past three decades, the development of consistent multicomponent thermodynamic databases has grown steadily, and several commercial software, such as Pandat^TM^^44^, Thermo-Calc^45^ and FactSage^46^, has become available. Although the CALPHAD approach has been well accepted in the design and development of advanced materials^21,47–51^, its full potential has not been fully released due to the low efficiency to explore the entire composition and temperature space of a multicomponent system. Here we need to emphasize that this trend is due to the limitation of the computational tools, but not the CALPHAD method.

In order to meet the increasing demand for massive calculations in the field of HEAs, an HTC tool in the frame of CALPHAD is developed and implemented in Pandat^TM^ ^44^. This CALPHAD-based HTC tool enables thousands of calculations in a defined compositional space to be performed automatically. Alloy compositions that satisfy user-defined criteria can then be identified through mining the thousands of simulated results. Here we would like to emphasize that the current HTC module is a combinatorial tool for both the high-throughput calculation and high-throughput screening. Its speed and efficiency are markedly improved and significantly advantageous over the conventional CALPHAD method.

### Sample preparation

To verify the reliability of the current HTC method, eight different alloys identified from thousands of compositions were fabricated via the arc-melting method with 99.9 weight percent (wt%) purity constituent elements. The nominal chemical compositions of these alloys are listed in Supplementary Table 1. To ensure chemical homogeneity, the ingot was melted at least six times before drop-casting.

### Microstructural characterizations

Scanning-electron (SEM), APT, and TEM were used for microstructural characterizations. Synchrotron XRD experiment was performed on the 11-ID-C beamline at the Advanced Photon Source (APS), Argonne National Laboratory (ANL). A beam energy of 111 keV (wavelength of 0.1173 Å) with a beam size of 0.5 × 0.5 mm was used. The samples for the SEM and XRD observations were polished, using the vibration polishing method after finer grinding with a 1200 grit SiC paper. The TEM specimen was prepared by a Ga+ focus ion beam (FIB) with a final milling voltage of 5 keV. The APT specimens were fabricated, employing the method described by Thompson et al.^52^. Equilateral triangular prism wedges were lifted out, using a Kleindiek MM3a, mounted on Si microtip array posts, sharpened using a 30 kV Ga+ ion beam, and cleaned using a 2 kV ion beam. The APT experiment was run, employing a CAMECA LEAP 4000XHR in a laser mode with 60 pJ, 10-ps-laser pulsed, a 30K-base temperature, 0.5% detection rate, and a pulse repetition rate that allowed for all ions to be collected in the mass spectrum. The APT results were reconstructed and analyzed using the CAMECA’s interactive visualization and analysis software (IVAS 3.8).

### Mechanical tests

Room and elevated-temperature compression tests of these eight alloys were carried out at the strain rate of 2 × 10^−4^ s^−1^, using the hydraulic Mechanical Testing System (MTS). The sample size for the compression tests is 3 mm in diameter and 6 mm in length. Each test was repeated three times.

### Neutron-scattering measurements

In situ neutron-scattering experiments were performed on selected discovered alloys at the Spallation Neutron Source (SNS), Oak Ridge National Laboratory (ORNL), to understand the deformation mechanisms and the order-disorder transition behavior. In situ neutron-diffraction experiments were conducted on Alloys 1 and 7 during their uniaxial compressive deformation on the VULCAN Engineering Materials Diffractometer, SNS, ORNL. The incident neutron beam with a beam size of 5 mm × 6 mm hits the cylindrical samples with a size of ϕ6 mm × 12 mm. To obtain a better d-spacing resolution, the high-resolution (HR) mode was chosen. After the measurements, the data were analyzed by the single-peak fitting method, using the event-based data reduction software, VULCAN Data Reduction and Interactive Visualization softwarE (VDRIVE)^53^. The PDFs of Alloys 1 and 7 were measured on the Nanoscale-Ordered Materials Diffractometer (NOMAD) instrument at SNS, ORNL^54^. The total neutron-scattering data were collected from RT to 1050 °C/1080 °C, using the ILL furnace installed on the NOMAD instrument. The samples were put into vanadium cans, and then heated at a ramp rate of 10 °C/min. under a vacuum environment (~10^−6^ mbar). At selected temperatures, the samples were held for 24 min. for data collection. The PDF, *G*(*r*), was obtained by a Fourier transformation of the structure function, *S*(*Q*), using a *Q*_max_ of 22 Å^−1^ ^55^,

3 $$G(r)=\frac{2}{\pi }\int Q[S(Q)-1]\,\sin (Qr){\rm{d}}Q$$

where Q is the scattering vector. The measured PDFs were fitted with the MC-calculated 2000 atoms structure, using the PDFgui software^56^.

### AIMD simulations

The atomic structure was predicted from AIMD simulations via the Vienna Ab Initio Simulation Package (VASP)^57,58^ in a canonical ensemble, i.e., a constant mole, volume, and temperature. Newton’s equations of motion were integrated, using the Verlet algorithm^59^ with a time step of 1 fs, and the atomic-configuration relaxation and temperature were controlled by a Nose thermostat^60^. Projector augmented-wave (PAW) potentials^61^ and the revised Perdew–Burke–Ernzerhof (PBE)^62^ gradient approximation to the exchange-correlation functional were used. We applied spin polarization, a single *k*-point, and an enhanced energy cutoff of 350 eV within a cubic supercell of 200 atoms. The liquid densities were determined by adjusting the cell volume so that the average pressure vanished in equilibrium at *T* = 1600 °C, yielding an atomic volume of 12.72 Å^3^/atom. Subsequently, hybrid Monte Carlo/molecular dynamics simulations^63^ were performed at this volume to facilitate sampling of the equilibrium ensemble.

The atomic structure in the liquid state can reveal the useful information about the preferred interatomic bonding that may impact the formation of the disordered solid solution during solidification^18,64^. The partial PDF gives the information about the probability of such bond formation by measuring the intensity of near-neighbor pairs against the total random distribution, and partial PDF [${g}_{ab}(r)$] was calculated, using

4 $${g}_{ab}(r)=\frac{V}{{N}_{a}{N}_{b}}\frac{1}{4\pi {r}^{2}}{\sum }_{i=1}^{{N}_{a}}{\sum }_{j=1}^{{N}_{b}}\langle \delta (|{r}_{ij}|-r)\rangle$$

where *V* is the volume of the supercell, *N*_*a*_ and *N*_*b*_ are the numbers of elements, *a* and *b*, $|{r}_{ij}|$is the distance between elements, *a* and *b*, and the bracket, < >, denotes the time average of different configurations.

### MC simulations

A lattice-gas model can describe the order-disorder phase transitions for the same and fixed lattice. In principle, sufficiently large lattices with sufficiently accurate interactions between the lattice points can accurately describe the real phase transitions that do not involve only changes of lattice types. The serious restrictions of lattice-gas models include the effects of lattice distortion and lattice vibration. Still, such models are found to successfully predict the order-disorder phase transitions in a number of conventional and high-entropy alloys (HEAs)^3,33,65–68^.

In this study, a lattice-gas model was constructed to describe the order-disorder phase transitions in a BCC lattice. We performed Metropolis MC simulations, using the nearest-neighbor interaction model for simplicity. In the nearest-neighbor interaction model, only the atomic interactions of the nearest neighbors between atoms are considered. The interaction parameters,${J}_{ij}$, are computed by

5 $${J}_{ij}=\varDelta {H}_{ij}/z$$

where $\varDelta {H}_{ij}$is the formation energy per atom of the solute pair, *i*, *j*, and *z* is the number of the nearest-neighbor bonds per atom. In the BCC structure, *z* = 8/2 = 4. Although each atom has 8 nearest-neighbor bonds, two atoms share them. Hence, the number has to be divided by 2. The formation energies are taken from the *Aflow* database of Curtarolo et al.^69^. The ${J}_{ij}$ parameters used in this study are tabulated in Table 2.

Applying numbers listed in Table 2, we performed three Metropolis MC simulations. Two are for Alloys 1 and 7 using 2000 atoms with 10^7^ MC steps, the third for the L2_1_ phase using 250 atoms with 10^6^ MC steps.

## Supplementary information

Supplementary Information

## Data Availability

The data that support the findings of this study are available from the corresponding author upon reasonable request.
