# Supplementary material for: High-throughput design of high-performance lightweight high-entropy alloys
Source: Nat Commun. 2021 Jul 15;12:4329. doi: 10.1038/s41467-021-24523-9 (PMC8282813; doi:10.1038/s41467-021-24523-9)
Supplement: Supplementary file 1 — Supplementary Information [file 41467_2021_24523_MOESM1_ESM.pdf]

Supplementary Information

High-throughput design of high-performance lightweight high-entropy alloys

Rui Feng[1,2], Chuan Zhang[3*], Michael C. Gao[4,5*], Zongrui Pei[4,6], Fan Zhang[3], Yan Chen[2], Dong Ma[7], Ke An[2], Jonathan D. Poplawsky[8], Lizhi Ouyang[9], Yang Ren[10], Jeffrey A. Hawk[4], Michael Widom[11], and Peter K. Liaw[1*]

[1]Department of Materials Science and Engineering, The University of Tennessee, Knoxville, TN 37996, USA;

[2]Neutron Scattering Division, Oak Ridge National Laboratory, Oak Ridge, TN 37831, USA;

[3]Computherm, LLC, 8401 Greenway Blvd. Suite 248, Middleton, WI 53562, USA;

[4]National Energy Technology Laboratory, 1450 Queen Ave SW, Albany, OR 97321, USA;

[5]Leidos Research Support Team, 1450 Queen Ave SW, Albany, OR 97321, USA;

[6]ORISE, 100 ORAU Way, Oak Ridge, TN 37830, USA;

[7]Neutron Science Platform, Songshan Lake Materials Laboratory, Dongguan, Guangdong, 523808, China;

[8]Center for Nanophases Materials Sciences, Oak Ridge National Laboratory, Oak Ridge, TN 37831, USA;

[9]Tennessee State University, Department of Physics and Mathematics, Nashville, TN 37209, USA;

[10]Advanced Photon Source, Argonne National Laboratory, Argonne, Illinois 60439, USA;

[11]Department of Physics, Carnegie Mellon University, Pittsburgh, PA 15213, USA.

[*] Corresponding authors: chuan.zhang@computherm.com, michael.gao@netl.doe.gov, and pliaw@utk.edu

Supplementary Figures

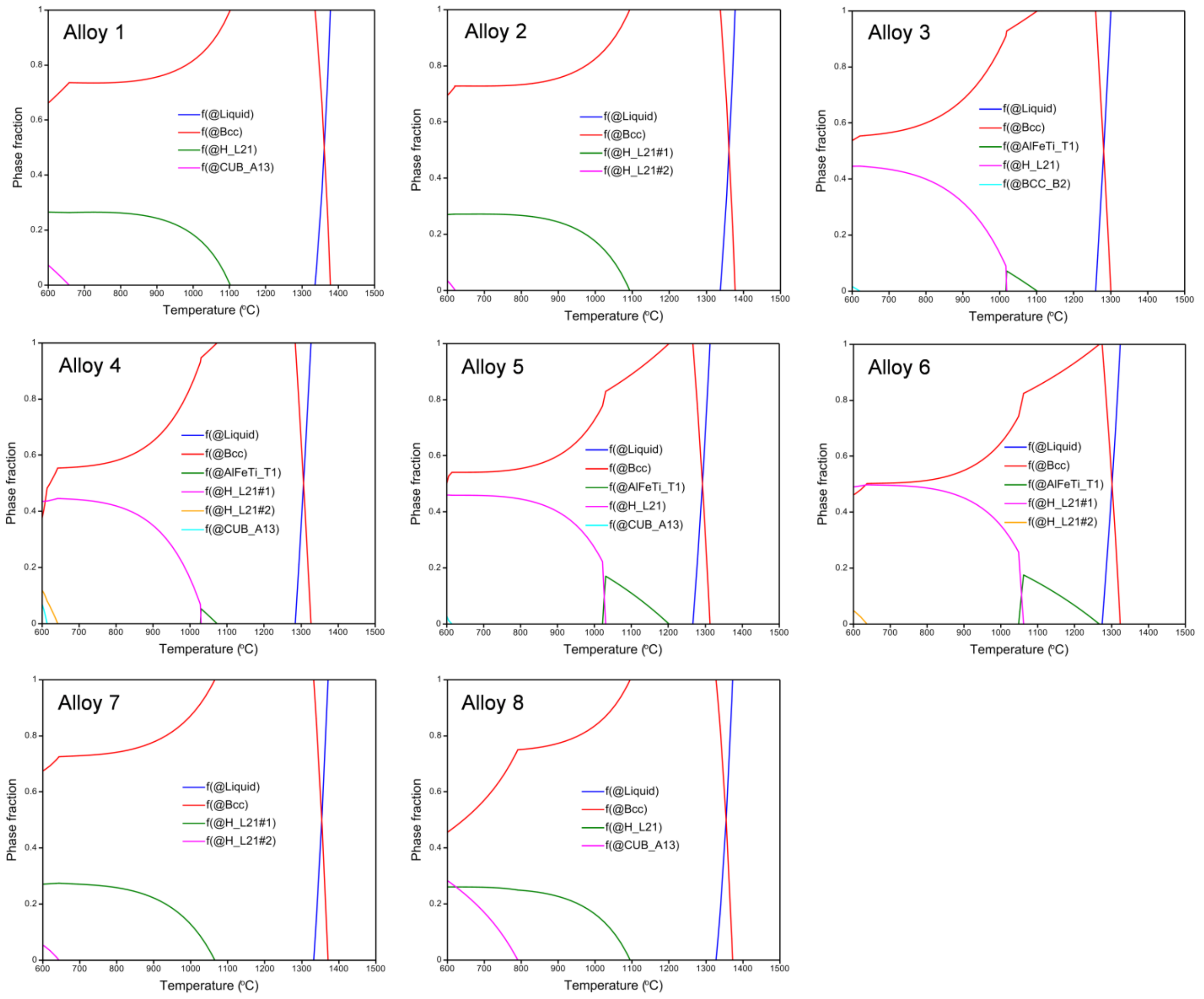

Supplementary Figure 1. Equilibrium phase-diagram calculations of the identified Alloys 1-8. Alloys 1 ($Al_{20}Cr_5Fe_{50}Mn_{20}Ti_5$), 2 ($Al_{25}Cr_5Fe_{50}Mn_{15}Ti_5$), 3 ($Al_{35}Cr_5Fe_{40}Mn_{10}Ti_{10}$), 4 ($Al_{30}Cr_{10}Fe_{35}Mn_{15}Ti_{10}$), 5 ($Al_{30}Cr_5Fe_{40}Mn_{15}Ti_{10}$), 6 ($Al_{30}Cr_5Fe_{45}Mn_{10}Ti_{10}$), 7 ($Al_{30}Cr_5Fe_{50}Mn_{10}Ti_5$), and 8 ($Al_{15}Cr_5Fe_{50}Mn_{25}Ti_5$).

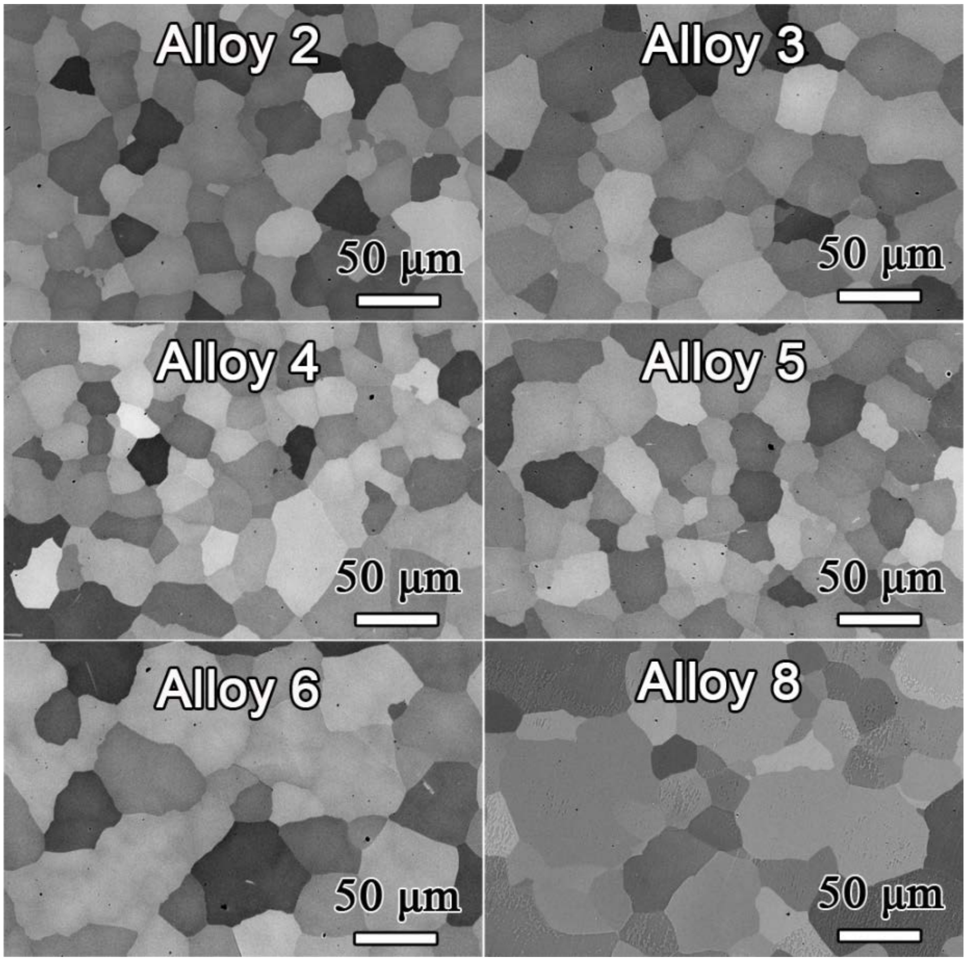

Supplementary Figure 2. SEM microstructures of the discovered alloys. BSE images of the designed Alloys 2-6 and 8 in their as-cast states, showing the equiaxed grains.

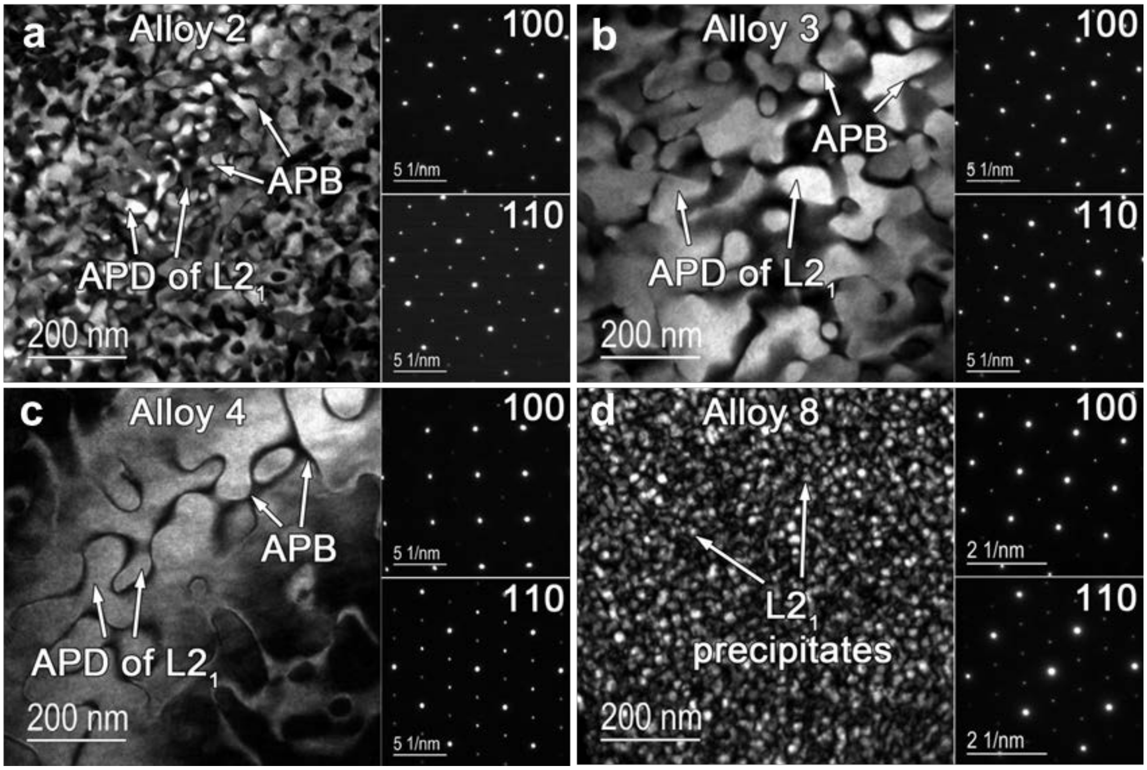

Supplementary Figure 3. TEM characterizations on Alloys 2-4 and 8. **a-d** The TEM DF images taken by the unique (111) reflection of the $L2_1$ phase and the corresponding selected-area diffraction (SAED) patterns along the [100] and [110] zone axes of Alloys 2 ($Al_{25}Cr_5Fe_{50}Mn_{15}Ti_5$), 3 ($Al_{35}Cr_5Fe_{40}Mn_{10}Ti_{10}$), 4 ($Al_{30}Cr_{10}Fe_{35}Mn_{15}Ti_{10}$), and 8 ($Al_{15}Cr_5Fe_{50}Mn_{25}Ti_5$) in their as-cast states, respectively.

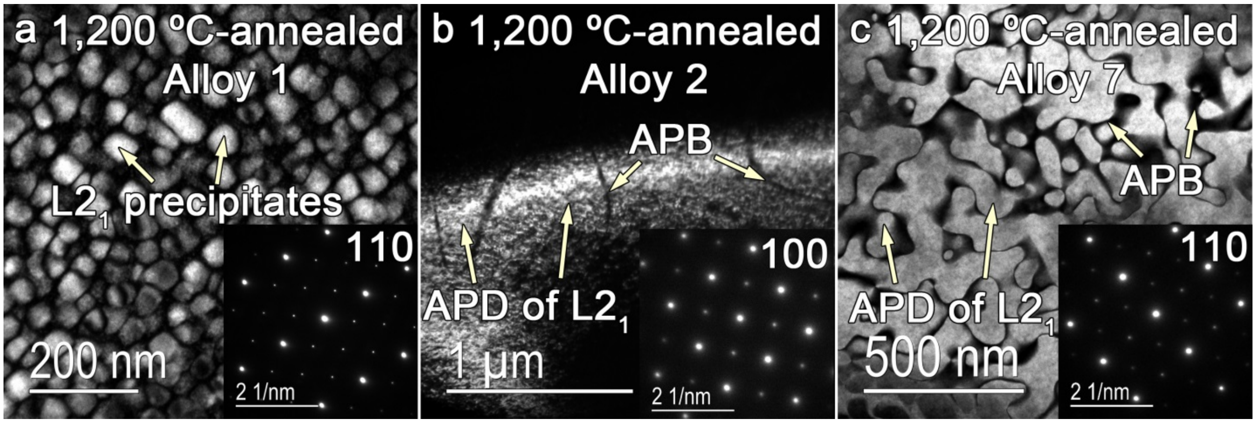

Supplementary Figure 4. TEM characterizations on Alloys 1, 2 and 7 after homogenizing at 1,200 ^{o}C for 30 minutes. **a-c** DF images of Alloys 1 ($Al_{20}Cr_5Fe_{50}Mn_{20}Ti_5$), 2 ($Al_{25}Cr_5Fe_{50}Mn_{15}Ti_5$), and 7 ($Al_{30}Cr_5Fe_{50}Mn_{10}Ti_5$), respectively, taken by the unique (111) or (100) reflections of the L2$_1$ phase and the corresponding SAED patterns along the [110] or [100] zone axis.

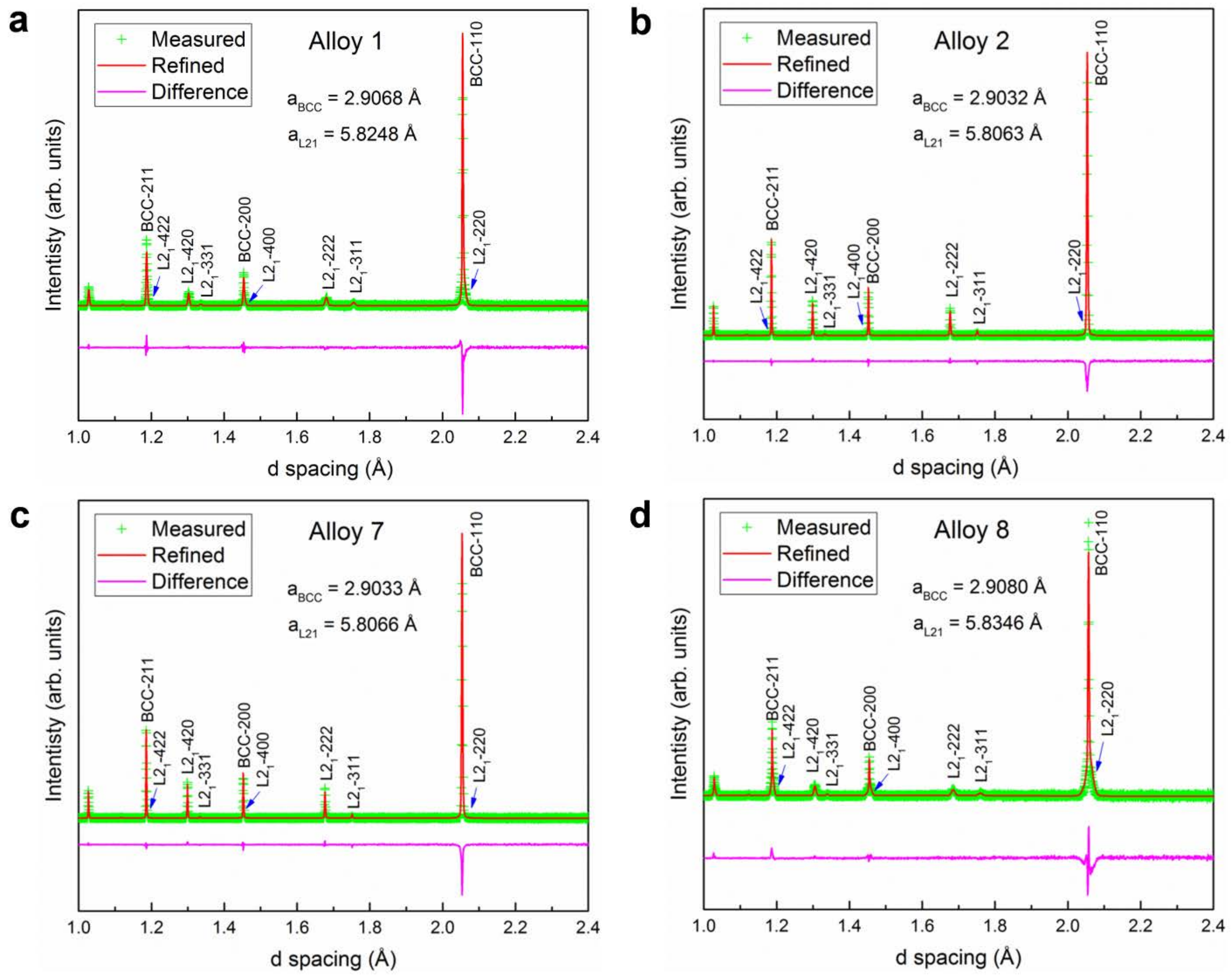

Supplementary Figure 5. GSAS Rietveld refinements on the neutron-diffraction results. a-d Alloys 1, 2, 7, and 8 at room temperature (RT), respectively. During the refinements, Alloys 2 and 7 were also treated as the BCC + $L2_1$ two-phase structure, and then we obtained the lattice misfits of Alloy 1 (0.192%), Alloy 2 (-0.002%), Alloy 7 (0.001%), and Alloy 8 (0.319%). The small lattice misfits of Alloys 2 and 7 indicate that these two alloys are more like a single $L2_1$ structure, as reflected by their refined very high $L2_1$'s volume fraction (~ 85%).

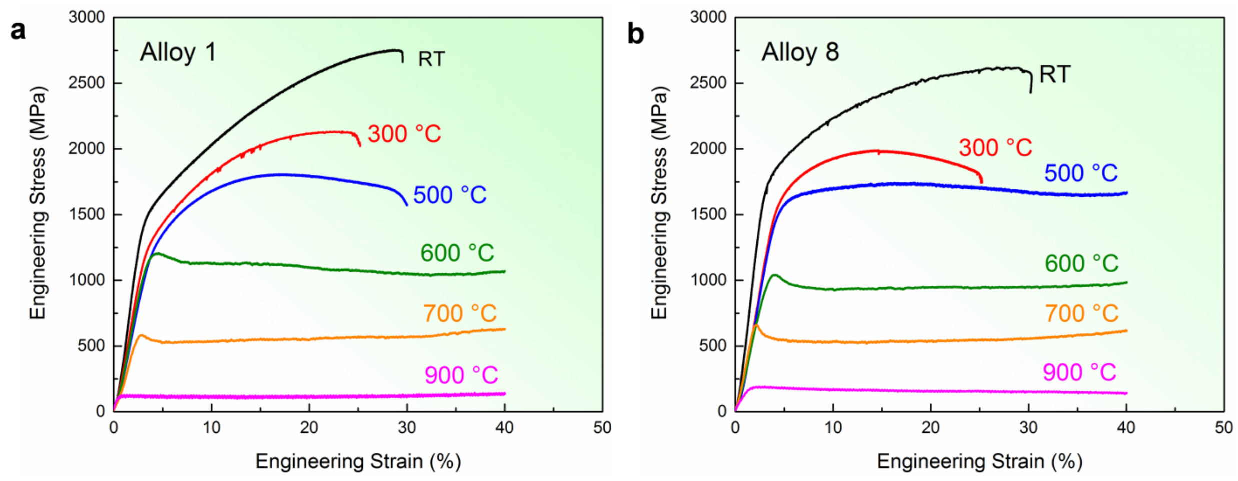

Supplementary Figure 6. High-temperature mechanical properties of Alloys 1 and 8. Compressive engineering stress-strain curves of Alloys 1 (**a**) and 8 (**b**) at elevated temperatures.

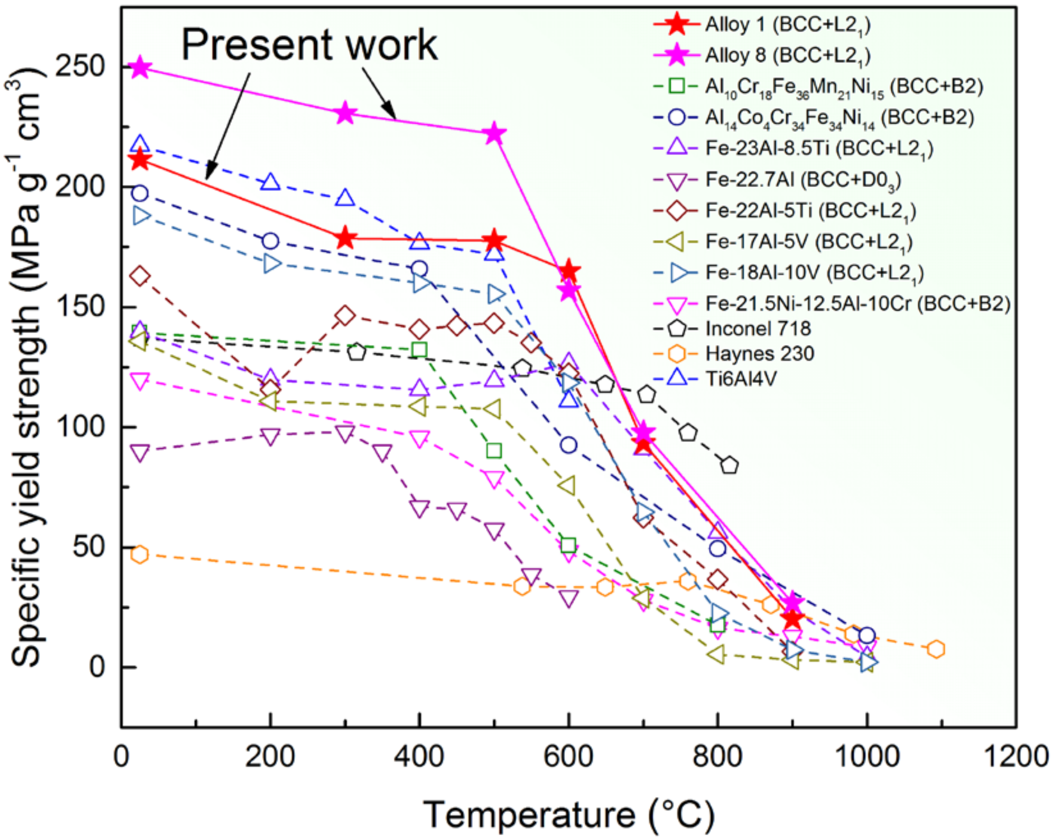

Supplementary Figure 7. High-temperature mechanical comparison in terms of specific yield strength. Comparison of specific yield strengths as a function of temperature between Alloys 1, 8, and other counterpart materials (all these BCC-base alloys are in their as-cast states)[1-5].

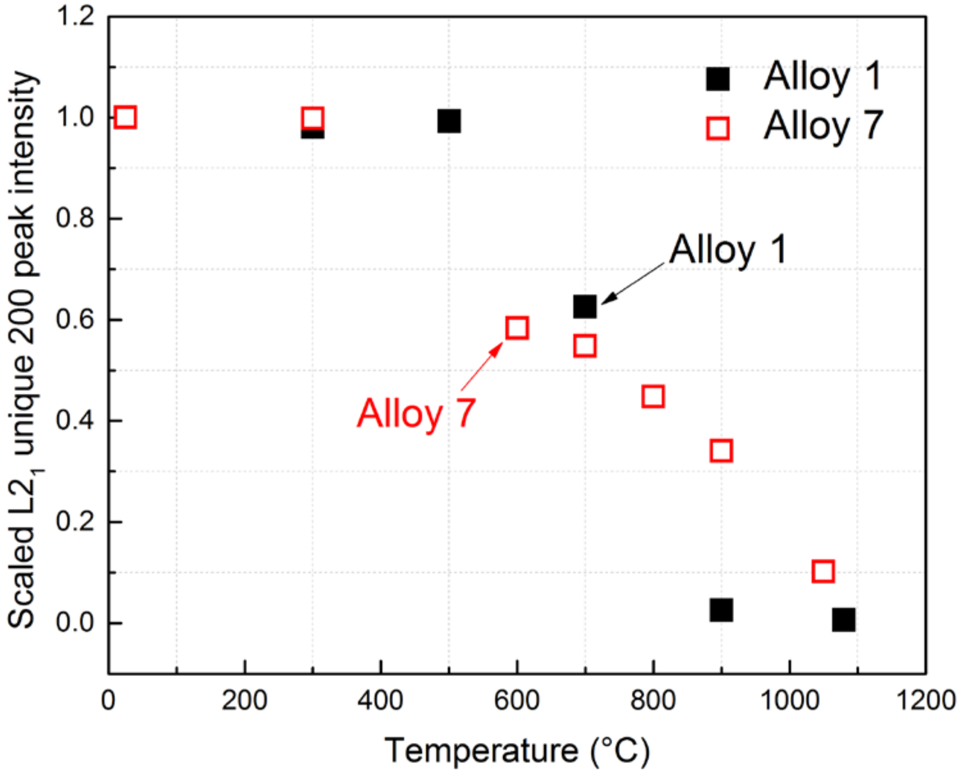

Supplementary Figure 8. Phase stability of the $L2_1$ phase in Alloys 1 and 7. The evolution of the $L2_1$ unique [200] neutron-diffraction peak intensity versus temperature of Alloys 1 and 7, scaled by the [200] intensity at RT, indicating the stable $L2_1$ phase in Alloy 7.

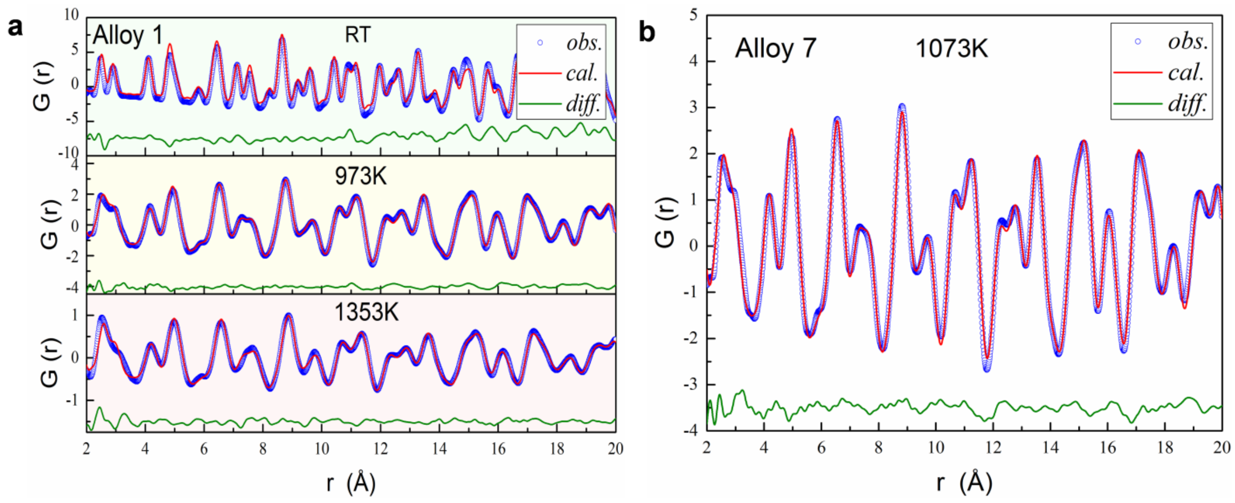

Supplementary Figure 9. Monte-Carlo (MC) superstructures fitted pair-distribution functions (PDFs). MC superstructures fitted neutron-scattering PDFs of Alloys 1 and 7 at different temperatures, suggesting the reliability of the MC 2,000-atom superstructures for both Alloys 1 and 7.

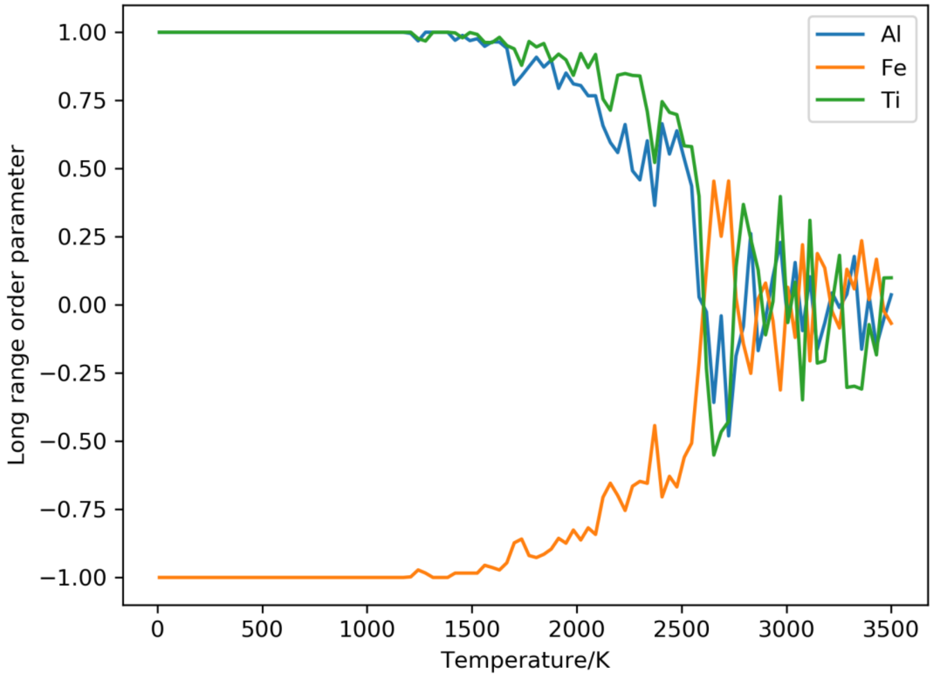

Supplementary Figure 10. Order-disorder transition of the Fe_2AlTi-type $L2_1$. The MC calculated LRO parameters versus the temperature of the Fe_2AlTi-type $L2_1$ structure.

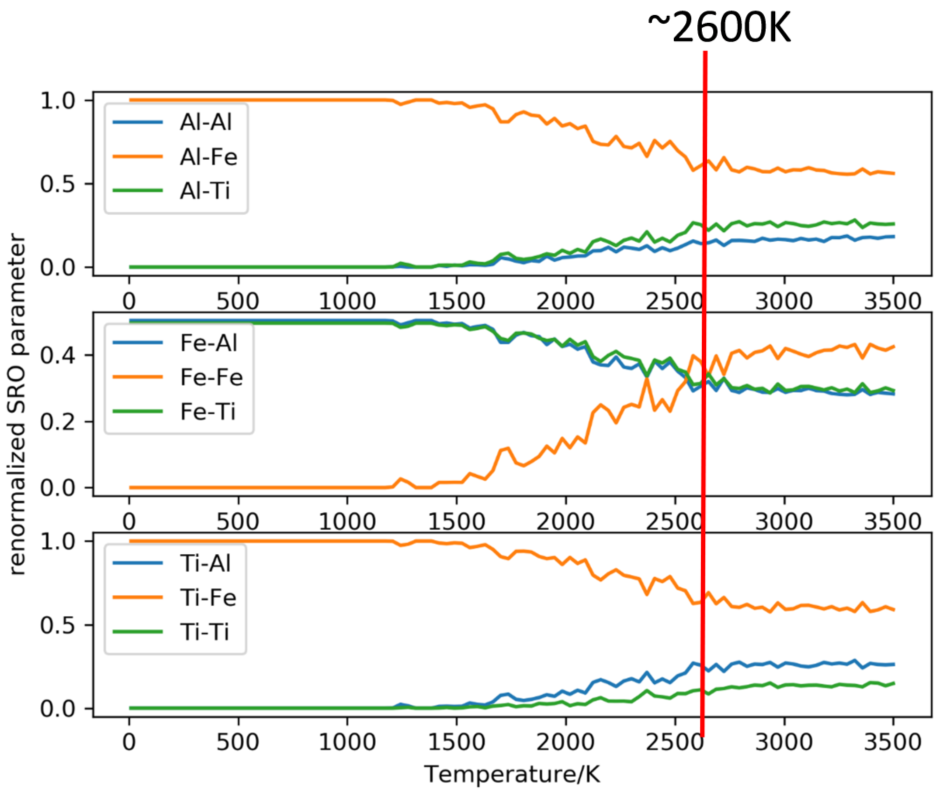

Supplementary Figure 11. MC-calculated SRO parameters evolution versus the temperature of the Fe_2AlTi-type $L2_1$. The first six SRO parameters of the Fe_2AlTi of the $L2_1$ structure. Above ~ 2,327 °C (2,600 K), all the renormalized SRO parameters approach the concentration fractions of the random state.

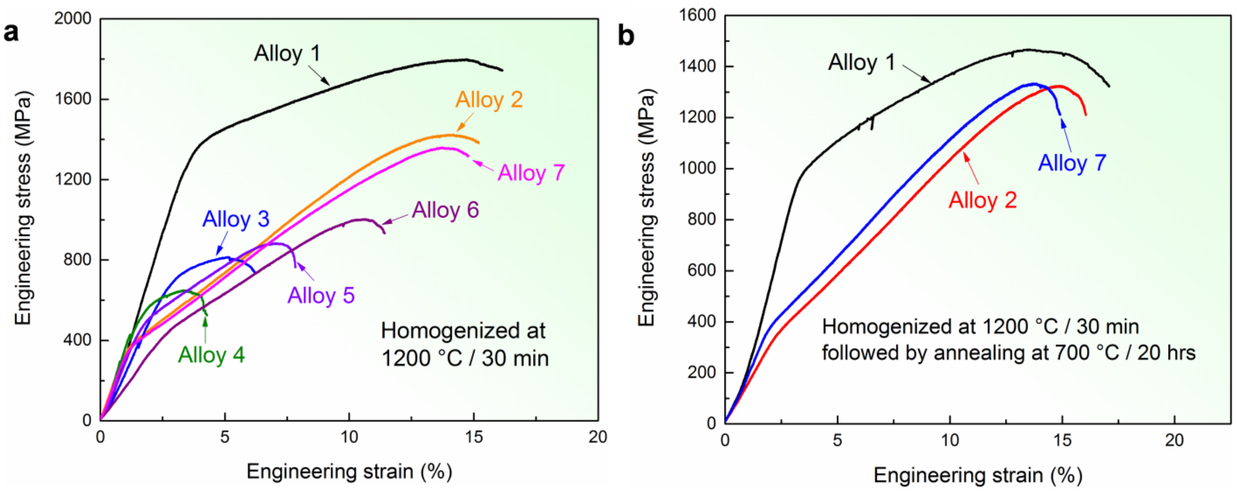

Supplementary Figure 12. Mechanical properties of the designed alloys after different heat treatments. a Compressive engineering stress-strain curves of Alloys 1-7 at RT after being homogenized at 1,200 °C for 30 minutes and **b** of Alloys 1, 2, and 7 after being homogenized at 1,200 °C for 30 minutes followed by 20 hours.

Supplementary Tables

Supplementary Table 1. Nominal chemical compositions of the identified eight alloys in atomic percent (at.%).

Alloys	Al (at.%)	Cr (at. %)	Fe (at. %)	Mn (at. %)	Ti (at. %)
Alloy 1	20	5	50	20	5
Alloy 2	25	5	50	15	5
Alloy 3	35	5	40	10	10
Alloy 4	30	10	35	15	10
Alloy 5	30	5	40	15	10
Alloy 6	30	5	45	10	10
Alloy 7	30	5	50	10	5
Alloy 8	15	5	50	25	5

Supplementary Table 2. Mechanical properties of Alloys 1-8 at RT, determined by compression tests.

Alloys	Yield stress, $\sigma_{0.2}$, MPa	Fracture stress, σ_f, MPa	Fracture strain, ε_f, %
Alloy 1	1,374 ± 23	2,666 ± 86	28 ± 2
Alloy 2	500 ± 12	2,385 ± 48	27 ± 1
Alloy 3	800 ± 11	1,170 ± 8	8 ± 0.2
Alloy 4	754 ± 32	1,194 ± 153	8 ± 1
Alloy 5	597 ± 6	1,564 ± 85	13 ± 1
Alloy 6	503 ± 8	1516 ± 1	13 ± 1
Alloy 7	514 ± 19	1,718 ± 149	16 ± 1
Alloy 8	1,642 ± 3	2,480 ± 111	29 ± 2

Supplementary Table 3. Yield strengths (MPa) of Alloys 1 and 8 at elevated temperatures, determined by compression tests.

	RT	300 °C	500 °C	600 °C	700 °C	900 °C
Alloy 1	1,374 ± 23	1,162 ± 10	1,154 ± 13	1,072 ± 25	607 ± 22	131 ± 5
Alloy 8	1,642 ± 3	1,517 ± 22	1,461 ± 30	1,032 ± 10	643 ± 16	176 ± 17

Supplementary Table 4. Conditional probabilities, $w_{\mu\nu}^{ij}$, determined by first-principles hybrid Monte Carlo/molecular dynamics for Alloy 1 at T = 1,600 K.

	Al	Cr	Fe	Mn	Ti
Al	0.11	0.06	0.54	0.25	0.04
Cr	0.23	0.04	0.55	0.17	0.02
Fe	0.21	0.06	0.46	0.19	0.07
Mn	0.25	0.05	0.49	0.18	0.03
Ti	0.16	0.02	0.68	0.12	0.02

Supplementary Notes

Supplementary Note 1. Precipitation-strengthening calculations

The precipitation strengthening can be divided into two categories, particle shearing or Orowan bowing[6]. In consideration of the observed small nanosized coherent $L2_1$ particles (~ 20 nm) from both TEM and APT, it is more likely that the shearing mechanism dominates the strengthening. For the coherent precipitation, coherency strengthening, $\Delta\sigma_{CS}$, modulus-mismatch strengthening, $\Delta\sigma_{MS}$, and order strengthening, $\Delta\sigma_{OS}$, contribute to the increase in the yield strength, which can be expressed as follows, respectively[7]:

$$\Delta\sigma_{CS} = M \cdot \alpha_{\varepsilon}\left(G\varepsilon_c\right)^{3/2}\left(\frac{rf}{0.5Gb}\right)^{1/2} \tag{1}$$

$$\Delta\sigma_{MS} = M \cdot 0.0055\left(\Delta G\right)^{3/2}\left(\frac{2f}{G}\right)^{1/2}\left(\frac{r}{b}\right)^{3m/2-1} \tag{2}$$

$$\Delta\sigma_{OS} = M \cdot 0.81\frac{\gamma_{apb}}{2b}\left(\frac{3\pi f}{8}\right)^{1/2} \tag{3}$$

where M is the Taylor factor, which equals 2.75 for the BCC structure and 3.06 for the FCC structure[8], α_{ε} = 2.6 (a constant)[9], $\varepsilon_c = 2/3(\Delta a/a)$[9] is the constrained lattice misfit, in which a is the lattice parameter and $\Delta a/a \approx 0.192\%$ (Supplementary Figure 5). G and ΔG are the shear moduli of the matrix and the shear modulus mismatch between the precipitates and matrix, respectively (G = 83 GPa for Alloy 1, obtained from the Kroner model[10] elastic-fitting results on the *in-situ* neutron-diffraction data during compression, ΔG is negligible). r is the average particle size ($r \approx$ 20 nm for Alloy 1). f is the volume fraction of the precipitates (f = 44% for Alloy 1). b is the Burgers vector (2.5174 Å). γ_{apb} is the antiphase boundary energy of the precipitates ($\gamma_{apb} \approx 0.2839$

J/m^2), which is calculated based on the bond energies listed in Table 2. As seen in Table 2, the energy per bond of Fe-Al and Fe-Ti are -0.047eV and -0.059eV, respectively. As an approximation, we consider the precipitates as the Fe_2AlTi type, so that we assume an average energy of J = -0.053eV as the bond energy of a pseudo binary. For a (110) plane with two atoms area, $A = \sqrt{2}a^2$ (a = 2.9068 Å), there are 4 bonds in total. Thus, the APB energy = $4J/A$ = 0.2839 J/m^2.

Substituting all the parameters into Eqs. (3-5), in Alloy 1, the strengthening contributions from coherency strengthening, $\Delta\sigma_{CS}$, modulus-mismatch strengthening, $\Delta\sigma_{MS}$, and order strengthening, $\Delta\sigma_{OS}$, are roughly determined to be ~ 224 MPa, ~ 0 MPa, and ~ 904 MPa, respectively. Since the value of $\Delta\sigma_{OS}$ is significantly larger than $\Delta\sigma_{CS} + \Delta\sigma_{MS}$, the precipitation strengthening ($\Delta\sigma_{OS}$) is mainly attributed to the ordering strengthening (904 MPa), which is close to the experimentally-determined strength differences between Alloys 1 and 2 (874 MPa), and between Alloys 1 and 7 (860 MPa).

Supplementary References

1. Krein R, Palm M, Heilmaier M. Characterization of microstructures, mechanical properties, and oxidation behavior of coherent A2+L21 Fe-Al-Ti. *J. Mater. Res.* **24**, 3412-3421 (2009).

2. Stallybrass C, Sauthoff G. Ferritic Fe–Al–Ni–Cr alloys with coherent precipitates for high-temperature applications. *Mater. Sci. Eng. A* **387-389**, 985-990 (2004).

3. Zhou Y, Jin X, Zhang L, Du X, Li B. A hierarchical nanostructured Fe34Cr34Ni14Al14Co4 high-entropy alloy with good compressive mechanical properties. *Mater. Sci. Eng. A* **716**, 235-239 (2018).

4. Shaysultanov DG, Salishchev GA, Ivanisenko YV, Zherebtsov SV, Tikhonovsky MA, Stepanov ND. Novel Fe36Mn21Cr18Ni15Al10 high entropy alloy with bcc/B2 dual-phase structure. *J. Alloys Compd.* **705**, 756-763 (2017).

5. Morris DG, Gunther S. Room and high temperature mechanical behaviour of a Fe3Al-based alloy with α-α″ microstructure. *Acta Mater.* **45**, 811-822 (1997).

6. Dieter GE, Bacon DJ. *Mechanical metallurgy*. McGraw-hill New York (1986).

7. Ardell A. Precipitation hardening. *Metall. Trans. A* **16**, 2131-2165 (1985).

8. Hosford WF. *Mechanical behavior of materials*. Cambridge University Press (2010).

9. Gerold V, Haberkorn H. On the critical resolved shear stress of solid solutions containing coherent precipitates. *Phys. Status Solidi B* **16**, 675-684 (1966).

10. Kröner E. Berechnung der elastischen Konstanten des Vielkristalls aus den Konstanten des Einkristalls. *Z. Phys.* **151**, 504-518 (1958).
